# Supplementary material for: Identification of photoperiod-induced specific miRNAs in the adrenal glands of Sunite sheep (Ovis aries)
Source: Front Vet Sci. 2022 Jul 22;9:888207. doi: 10.3389/fvets.2022.888207 (PMC9354845; doi:10.3389/fvets.2022.888207)
Supplement: Supplementary file 1 [file Data_Sheet_1.ZIP › Supplementary file/miRDeep2 file.zip!/miRDeep2 file/Know_Structure/SPLP42A2/oar-mir-29a.pdf]

miRBase precursor : oar-mir-29a
Total read count : 138114
oar-miR-29a read count : 138089
remaining reads : 25

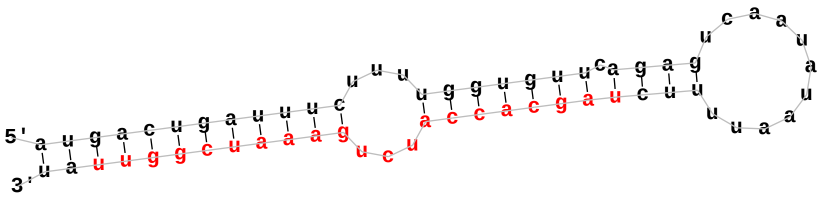

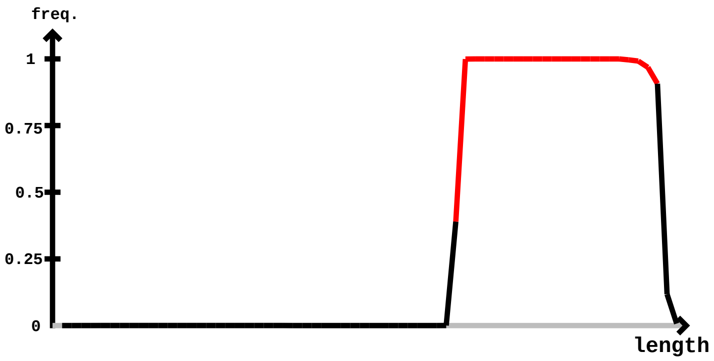


```
                                       oar-miR-29a
5'- augacugauuucuuuugguguucagagucaauauaauuuucuagcaccaucugaaaucgguuau   -3'    exp
    ((((((((((((...(((((((.((((...........)))))))))))...))))))))))))    reads      mm         sample
    .ugacugauuucuuuugguguu..........................................    2          0          seq
    .ugacugauuucuuuugguguuc.........................................    6          0          seq
    ...acugauuucuuuugguguu..........................................    1          0          seq
    ...acugauuucuuuugguguuc.........................................    16         0          seq
    .......................................ucuagcaccaucugaaaucgg....    10         0          seq
    .......................................ucuagcaccaucugaaaucggu...    13         0          seq
    .......................................ucuagcaccaucugaaaucgguu..    7          0          seq
    .......................................ucuagcaccaucugaaaucgguua.    1          0          seq
    ........................................cuagcUccaucugaaau.......    1          1          seq
    ........................................Uuagcaccaucugaaau.......    1          1          seq
    ........................................cuagcaccaucugaaau.......    7          0          seq
    ........................................cuagcaccaucugaaauc......    96         0          seq
    ........................................cuagcaccaucugaaauA......    7          1          seq
    ........................................cuagcaccauUugaaaucg.....    5          1          seq
    ........................................cuagcaccaucugaaaucg.....    132        0          seq
    ........................................cuagcaccaucugaaaucgg....    500        0          seq
    ........................................cuagcaccauUugaaaucgg....    3          1          seq
    ........................................cuagcaccaucugaaaucggu...    2265       0          seq
    ........................................cuagcaccauUugaaaucggu...    31         1          seq
    ........................................cuagcaccauUugaaaucgguu..    188        1          seq
    ........................................cuagcaccaucugaaaucgguu..    49705      0          seq
    ........................................cuagcaccaucugaaaucgguua.    835        0          seq
    ........................................cuagcaccauUugaaaucgguua.    2          1          seq
    ........................................cuagcaccaucugaaaucgguuau    45         0          seq
    .........................................uagcaccaucugaaauc......    340        0          seq
    .........................................Aagcaccaucugaaauc......    2          1          seq
    .........................................uaUcaccaucugaaauc......    2          1          seq
    .........................................uagcacUaucugaaauc......    1          1          seq
    .........................................uagcaccaucugaaGuc......    2          1          seq
    .........................................uagcaccaucugaaauA......    6          1          seq
    .........................................uagcaccaCcugaaauc......    4          1          seq
    .........................................uagcaccaucugaaaGc......    1          1          seq
    .........................................uagcaccaucugaaauU......    21         1          seq
    .........................................uagcaccaucugaaaucg.....    432        0          seq
    .........................................uagcaccaucugaCaucg.....    1          1          seq
```

oar-miR-29a

```
augacugauuucuuuuggugguucagagucaauauaauuuucuagcaccaucugaaaucgguuau
..........................................uagcaccauUugaaaucg.....    44       1        seq
..........................................uagcaccauUugaaaucgg....    201      1        seq
..........................................uagcaccaucugaaaucgg....    2529     0        seq
..........................................uagcaccaucugaaaucggu...    5465     0        seq
..........................................uagcaccauUugaaaucggu...    698      1        seq
..........................................uagcaccaucugaaaucgguu..    56622    0        seq
..........................................uagcaccauUugaaaucgguu..    2453     1        seq
..........................................uagcaccauUugaaaucgguua.    682      1        seq
..........................................uagcaccaucugaaaucgguua.    13676    0        seq
..........................................uagcaccaucugaaaucgguuau    942      0        seq
..........................................uagcaccauUugaaaucgguuau    38       1        seq
...........................................agcaccaucugaaaucg.....    1        0        seq
...........................................agcaccaucugaaaucgg....    1        0        seq
...........................................agcaccaucugaaaucggu...    4        0        seq
...........................................agcaccauUugaaaucgguu..    2        1        seq
...........................................agcaccaucugaaaucgguu..    48       0        seq
...........................................agcaccaucugaaaucgguua.    13       0        seq
............................................gcaccaucugaaaucgg....    1        0        seq
............................................gcaccaucugaaaucggu...    1        0        seq
............................................gcaccaucugaaaucgguua.    2        0        seq
```
